# Redefining Pediatric SCIWORA: A Systematic Review of the Literature on Clinical Patterns, Imaging Profiles, and Management Insights

**DOI:** 10.3390/jcm14176338

**Published:** 2025-09-08

**Authors:** Davide Palombi, Marco Galeazzi, Paolo Brigato, Sergio De Salvatore, Timothée De Saint Denis, Luca Massimi, Gianpiero Tamburrini, Leonardo Oggiano

**Affiliations:** 1 Department of Pediatric Neurosurgery, Fondazione Policlinico Agostino Gemelli IRCCS, Università Cattolica del Sacro Cuore, 00168 Roma, Italy; davide.palombi01@icatt.it (D.P.); mgaleazzi@live.it (M.G.); luca.massimi@policlinicogemelli.it (L.M.); gianpiero.tamburrini@policlinicogemelli.it (G.T.); 2 Department of Neuroscience, Università Cattolica del Sacro Cuore, 00168 Roma, Italy; 3 Research Unit of Orthopaedic and Trauma Surgery, Department of Medicine and Surgery, Università Campus Bio-Medico di Roma, Via Alvaro del Portillo, 00128 Roma, Italy; 4 Orthopedic Unit, Department of Surgery, Bambino Gesù Children’s Hospital, 00165 Rome, Italy; leonardo.oggiano@opbg.net; 5 Department of Pediatric Orthopedic and Reconstructive Surgery, Armand Trousseau Hospital, AP-HP, Sorbonne University, 75012 Paris, France; timothee.saintdenis@aphp.fr

**Keywords:** SCIWORA, pediatric trauma, spinal cord

## Abstract

**Objectives:** Among the spectrum of spinal injuries, Spinal Cord Injury Without Radiographic Abnormality (SCIWORA) occupies a unique and challenging position. SCIWORA presents diagnostic and therapeutic challenges due to its broad clinical and radiological heterogeneity. While most children recover favorably with conservative treatment, a subset may require surgery based on imaging findings. The findings underscore the need for standardized diagnostic criteria, MRI-based classification systems, and evidence-based treatment algorithms to improve consistency in care and long-term neurological outcomes. **Methods:** A systematic search of PubMed, Cochrane, Scopus, and Embase databases was performed through June 2025 following PRISMA guidelines. Inclusion criteria encompassed studies of pediatric SCIWORA (age < 18 years) reporting demographics, clinical and radiological features, management, and outcomes. **Results:** Sixty studies encompassing a total of 848 pediatric patients were included. The mean patient age was 9.33 years (±2.52), with a slight male predominance. The most common trauma mechanisms were road traffic accidents (40.3%), sports injuries (22%), and falls (18.8%). MRI findings were available in 399 cases: 46% had intraneural lesions (Type IIb), 39% showed no abnormality on MRI (Type I, or “real SCIWORA”), 9% had combined lesions (Type IIc), and 6% had extraneural abnormalities (Type IIa). Neurological severity at presentation was primarily ASIA Grade A (46.25%), but follow-up data showed substantial improvement, with ASIA E (normal function) increasing to 49.78%. Overall, 66.2% of patients experienced neurological improvement, while 33.8% remained stable. Conservative treatment was employed in 95.41% of cases. Only 4.59% underwent surgery, which was typically reserved for MRI-positive lesions demonstrating spinal instability or compression. **Conclusions:** Pediatric SCIWORA remains an uncommon but potentially devastating injury, with an outcome highly dependent on MRI findings and initial neurological status. This systematic review aims to clarify the contemporary understanding of pediatric SCIWORA, delineating “real” SCIWORA from other SCIWORA-like entities, and synthesizing the latest evidence regarding epidemiology, mechanisms, clinical presentation, MRI findings, and management in children.

## 1. Introduction

Traumatic spinal cord injury in children is a rare but potentially devastating event, often resulting in significant lifelong sequelae with profound social and psychological impact. Among the spectrum of spinal injuries, Spinal Cord Injury Without Radiographic Abnormality (SCIWORA) occupies a unique and challenging position. Initially defined in 1982 by Pang and Wilberger, SCIWORA described cases of traumatic myelopathy in children who presented with objective neurological deficits after trauma, but with no evidence of vertebral fracture or dislocation on X-rays or computed tomography (CT) [1]. With the subsequent advent and widespread use of magnetic resonance imaging (MRI), the concept and boundaries of SCIWORA have evolved—now encompassing a heterogeneous group of injuries and sparking debate over its precise definition [2,3,4]. The reported incidence of SCIWORA among pediatric spinal cord injuries varies widely, ranging from 13% to 42%, due in part to differences in imaging protocols and diagnostic criteria. Young children are especially vulnerable, attributable to unique anatomical and biomechanical features of the immature spine: greater ligamentous laxity, shallow and horizontally oriented facet joints, incomplete ossification, and a relatively large head-to-body ratio. These features allow for a high degree of vertebral column motion and “stretchability,” potentially surpassing the physiological limits of the spinal cord itself, thus predisposing to spinal cord damage in the absence of radiographically detectable skeletal injury [5,6,7]. Despite advances in imaging, uncertainty persists regarding the nosology of SCIWORA. While MRI is now considered mandatory for all suspected cases, studies reveal that a considerable proportion (“real” SCIWORA) show no abnormality even on high-resolution MRI, raising questions about the underlying pathophysiology and whether other conditions might be misclassified under this label, such as spinal cord concussion or transient neuropraxia [8,9,10,11]. Conversely, the identification of subtle intramedullary or extraneural lesions on MRI has led some authors to advocate for alternative nomenclature and more granular classification schemes [12,13,14]. Clinically, pediatric SCIWORA displays a broad spectrum: from transient and rapidly resolving neurological deficits to severe, permanent paralysis. The timing of symptom onset is variable, with some patients exhibiting delayed deficits, further complicating diagnosis [15,16,17,18,19]. MRI findings, when present, are highly heterogeneous and include cord edema, hemorrhage, contusion, or soft tissue injury patterns; however, the prognostic implications of these imaging features are incompletely understood. Several studies and consensus statements, including those by international neurosurgical societies, have attempted to refine diagnostic criteria and propose severity classifications, yet a universally accepted definition remains elusive [20,21]. Given the ongoing ambiguities in definition, diagnosis, and optimal management, the primary aim of this systematic review is to clarify the contemporary understanding of pediatric SCIWORA. Specifically, this review aims to establish what truly constitutes SCIWORA in the era of advanced imaging, delineating “real” SCIWORA from other SCIWORA-like entities, and synthesizing the latest evidence regarding epidemiology, mechanisms, clinical presentation and course, and MRI findings in children [22,23,24].

## 2. Materials and Methods

### 2.1. Search Strategy and Eligibility Criteria

A systematic literature search was performed following PRISMA guidelines. Electronic databases (PubMed, Cochrane, Scopus, Embase) were searched up to June 2025, using terms including “SCIWORA,” “spinal cord injury without radiographic abnormality,” “spinal cord injury without radiological abnormality,” “spinal cord injury with normal radiographs,” “MRI-negative spinal cord injury,” “pediatric,” “children,” and “child,”. Studies were included if they: (1) enrolled patients aged < 18 years, (2) described cases of acute spinal cord injury following trauma, (3) had no radiographically visible fractures or dislocations on plain films/CT, and (4) provided data on demographics, mechanism, imaging, clinical presentation, management, or outcome. Reviews, animal studies, and case series/individual cases lacking sufficient detail were excluded.

### 2.2. Data Collection Process

After the removal of duplicates, an initial screening of titles and abstracts was performed independently by two authors (D.P. and M.G.). Articles without an available abstract or lacking the relevant data were excluded. A full-text review of the remaining studies was then conducted to determine eligibility. In cases of disagreement between the reviewers, a senior author (L.O.) was consulted to reach a final decision.

### 2.3. Risk of Bias Assessment

The risk of bias of the included studies was systematically assessed using the Joanna Briggs Institute (JBI) critical appraisal tools [25]. Specifically, the JBI checklist for case reports and case series was applied, according to the study design. Two reviewers (D.P and M.G) independently evaluated each study, and disagreements were resolved through discussion or consultation with a senior author (L.O).

### 2.4. Data Extraction and Synthesis

Data extracted included: study design; sample size; age and sex; injury mechanism; spinal level(s) involved; American Spinal Injury Association Impairment Scale (ASIA) at admission and discharge; management approach; and outcomes (neurological improvement, complications, mortality).

MRI findings were classified according to the system proposed by Boese and colleagues [14]. Four imaging patterns were distinguished: Type I, with no detectable abnormalities; Type IIa, showing extraneural abnormalities (e.g., ligamentous or disc lesions); Type IIb, characterized by intraneural abnormalities such as cord edema, hemorrhage, or contusion; and Type IIc, with combined intra- and extraneural abnormalities. This classification was applied to improve comparability across studies and to explore potential correlations between imaging patterns and clinical outcomes.

### 2.5. Data Analysis

Data were summarized descriptively. For categorical variables (e.g., mechanism, level, outcome), frequencies and percentages were calculated. For continuous variables (e.g., age), means and standard deviations were recorded. Tables were prepared to present patient demographics, injury characteristics, MRI findings, management strategies, and outcomes.

## 3. Results

### 3.1. Study Selection

**Figure 1:**
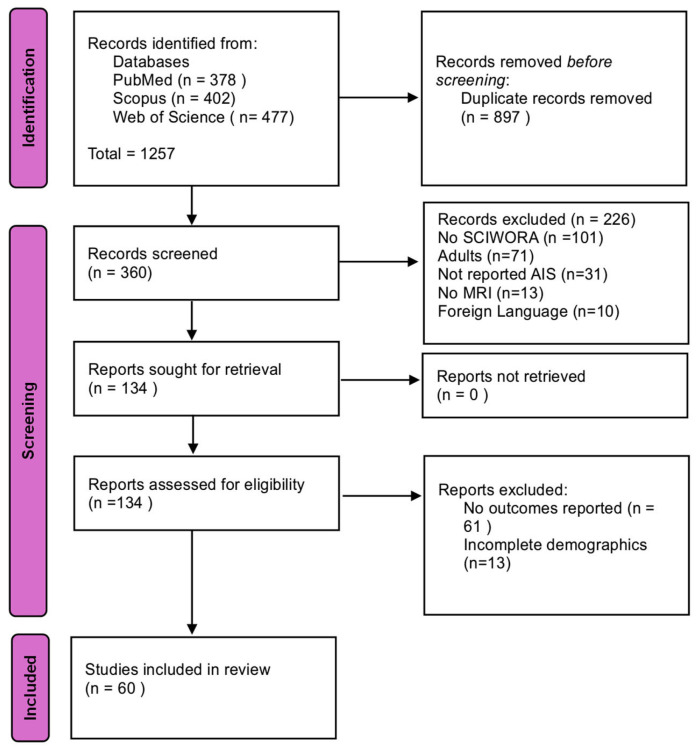
PRISMA flowchart diagram.

After removal of duplicates, 1257 articles were identified for title/abstract screening. 360 reports underwent full-text review, of which 60 met all criteria and were included (Figure 1 PRISMA flowchart).

### 3.2. Risk of Bias Assessment Results

Most of the included studies fulfilled the main JBI criteria, with clear objectives, adequate description of populations, and consistent outcome reporting. Only a minority lacked detailed follow-up information or explicit inclusion criteria. Overall, the methodological quality was acceptable and allowed a reliable descriptive synthesis of the available evidence. Results are summarized in Supplementary Tables S1 and S2.

### 3.3. Study Characteristics and Population Overview

A total of 848 pediatric patients diagnosed with SCIWORA were analyzed from 60 included studies. As shown in Table 1, the mean age was 9.33 years (±2.52), with a slight male predominance (54% male, 46% female).

### 3.4. Trauma Mechanisms and MRI Findings

**Figure 2:**
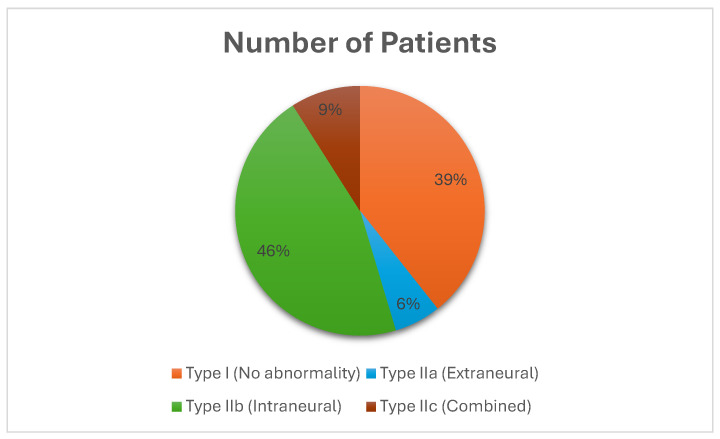
This pie chart illustrates the distribution of MRI findings in 848 pediatric patients diagnosed with SCIWORA. Only 399 MRI results were reported. Intraneural abnormalities (Type IIb) were the most frequent finding (46%), followed by cases with no radiological abnormalities (Type I, 39%), referred to as “real SCIWORA.” Combined lesions (Type IIc) accounted for 9%, and extraneural abnormalities (Type IIa) were the least frequent (6%). These results underscore the heterogeneity of radiological patterns and the diagnostic value of MRI in guiding prognosis and management.

Road traffic accidents (RTAs) were the predominant trauma mechanism, accounting for 40.3% of cases, followed by sports-related injuries (22%), falls (18.8%), and other causes (13.8%). MRI findings were reported for 399 patients. Intraneural abnormalities (Type IIb) represented the most common MRI lesion (46%), followed by “real SCIWORA” cases with no abnormalities (Type I, 39%). Combined lesions (Type IIc, 9%) and extraneural abnormalities (Type IIa, 6%) were less frequent, as shown in Figure 2.

### 3.5. Neurological Outcomes

**Figure 3:**
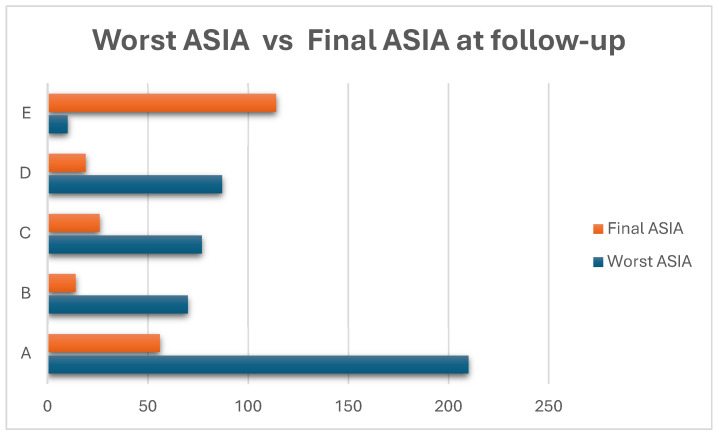
This horizontal bar chart illustrates the distribution of ASIA grades at the time of worst neurological impairment during the hospitalization (blue bars) and at final follow-up (red bars) in 454 pediatric SCIWORA patients. At presentation, nearly half of the patients (46.25%) were classified as ASIA A, indicating complete spinal cord injury. However, at follow-up, the number of patients remaining in ASIA A decreased substantially to 24.45%, suggesting partial or complete neurological recovery in a significant portion. Conversely, the number of patients with normal neurological function (ASIA E) increased from only 4.54% at baseline to nearly 50% at follow-up. The remaining ASIA grades (B, C, and D) followed a similar trend, with decreases in severe grades and increases in less severe or normal findings.

Table 2 and Figure 3 show the neurological initial and final status of the reported cases. Neurological outcomes were assessed in 454 patients using the ASIA impairment scale. At presentation, ASIA Grade A was the most frequent classification (46.25%), indicating complete spinal cord injury. However, substantial neurological improvement was observed at follow-up, with ASIA Grade A decreasing to 24.45% and ASIA Grade E (normal neurological function) increasing significantly from 4.54% to 49.78%. Overall, 66.2% of patients showed neurological improvement, while 33.8% remained stable.

### 3.6. Conservative Management and Surgical Treatment

Out of 848 patients, 95.41% were treated conservatively with medical therapy, while 39 (4.59%) underwent surgical intervention primarily for cervical lesions (Table 3). Types of surgical procedures included halo-gravity traction, decompressive laminectomy, fusion, and stabilization. Controversially, three patients underwent lysis of the filum terminale for associated tight filum, highlighting ongoing ambiguity in surgical indications for SCIWORA cases [63].

## 4. Discussion

The original term, as coined by Pang and Wilberger, refers to traumatic myelopathy with normal plain radiographs and CT scans. With MRI now routine, the landscape is more complex: of 848 pediatric cases aggregated in our review, 39% had “real SCIWORA” (normal MRI, Type I), while the majority disclosed some form of MRI-detectable abnormality (intraneural 46%, extraneural 6%, combined 9%). Such variability reflects the ongoing dispute in the literature, where SCIWORA retains its significance and complexity, particularly in pediatrics.

Some authors now reserve “real SCIWORA” solely for cases entirely negative on X-ray, CT, and MRI, while “SCIWORA-like” encompasses patients with neural or extraneural MRI changes absent on earlier imaging. As multiple consensus statements and recent meta-analyses highlight, the lack of a uniform definition creates inconsistent reporting, complicates inter-study comparisons, and directly impacts management strategies and prognostication. This systematic review underscores this, with a substantive minority (39% overall) qualifying as “real SCIWORA,” nearly reflecting the 43% of patients reported in previous reviews [14].

### 4.1. Mechanisms of Trauma, Clinical Presentation, and MRI

Out of 848 patients, trauma mechanism analysis (Table 1; Figure 2) reported sports injuries (41%), motor vehicle accidents (RTA; 22%), and falls from height (19%) as the prevalent causes, closely aligning with robust epidemiological studies [6,10,48].

Age also plays a central role: the mean age in the cohort is 9.33 ± 2.52 years, but mechanisms and injury levels differ with age. Young children (especially those < 8 years) have a higher proportion of high cervical and thoracic injuries and are more likely to suffer from motor vehicle accidents and falls. In contrast, older children and adolescents are predominantly affected by sports trauma. Consistent with the literature, delayed onset of neurological symptoms after injury (defined as >6 h post-trauma) was noted in 18% of the reported cases.

Crucially, MRI has shifted the diagnostic paradigm: only 39% of reported SCIWORA cases now have normal scans, with the remainder showing varying patterns of cord edema, hemorrhage, or extraneural injury. 60.6% of the patients reported had an MRI positivity, based on the MRI classification proposed by Boese et al., which robustly stratifies risk [14].

Clinical correlation is strong: Type I cases in this review (including the 32-patient series by Freigang et al.) mostly had full neurologic recovery, affirming Boese’s and Carroll’s findings that normal MRI predicts excellent prognosis in pediatric SCIWORA. Conversely, Type IIb/IIc patients, particularly those with intramedullary hemorrhage, had persistently worse outcomes [14,45,46,47,48,51,56].

Beyond these prognostic categories, several radiological studies have emphasized the importance of more detailed MRI assessment in pediatric SCIWORA [61,64]. Advanced analyses have shown that not only the presence, but also the extent of cord involvement, including lesion length and maximum cross-sectional area, strongly correlates with neurological recovery [61]. Farrell et al. further underlined that the choice of MRI sequences and timing of acquisition may critically influence the detection of subtle abnormalities, while at the same time, issues of sedation in young children remain a practical challenge for protocol optimization [64].

### 4.2. ASIA Outcomes, Neurological Improvement Rates, and Comparative Analysis

Neurological severity as measured by the American Spinal Injury Association Impairment Scale at admission strongly influences both short- and long-term outcomes. Across both our data and the literature, the spectrum at presentation ranges from complete injury (ASIA A) through incomplete (ASIA B, C, D), with a higher proportion of complete lesions in younger children [34,61]. Notably, in this series, two-thirds of patients achieved at least one-grade improvement on the ASIA at latest follow-up, a finding that closely mirrors the 39–67% improvement rates cited in large series and meta-analyses [31,54,62].

The literature and results reported here agree: the best prognoses are observed in children with normal MRIs or isolated cord edema, especially those with incomplete initial deficits [5,29,62,65]. In contrast, complete injuries (ASIA A) and those with cord hemorrhage rarely show substantial improvement, underscoring the value of early and precise prognostication. However, long-term sequelae, including neurogenic bladder and progressive spinal deformities, are common, particularly in those with poor initial grade [14,22].

### 4.3. Lack of Consensus and General Management Approaches

Management of pediatric SCIWORA remains equally controversial and poorly standardized. While most patients are managed conservatively with immobilization and activity restriction, the optimal duration and modality of immobilization, the role of surgery in specific subtypes, and even the potential use (or harm) of high-dose corticosteroids are all debated. Early literature advocated for prolonged rigid immobilization to prevent the recurrence of injury, yet more recent data suggest that individualized therapy informed by MRI may be more appropriate [20,44].

Conservative management, characterized by immobilization and rehabilitation, was predominant in this study (95.41%), while in only 4.59% of cases was surgical intervention required. This aligns with most major series affirming strict immobilization and “watchful waiting” in the absence of MRI evidence for instability or extraneural compression [14,62].

Steroid administration was given in 34% of cases, primarily in those with incomplete injuries and acute onset, reflecting ongoing variability and lack of clear consensus [38]. No statistically significant correlation was reported in the literature between steroid use and neurological recovery, as shown in recent systematic recommendation [14,63], which suggest steroids cannot be considered standard care in pediatric SCI.

Recent reviews and clinical practice guidelines further highlight the lack of strong evidence supporting corticosteroid use in acute pediatric SCI [14,63,65,66]. Dudney and Sherburn emphasized that most available data derive from case reports and series, with inconsistent reporting and methodological limitations, precluding definitive conclusions on efficacy [10]. Similarly, the most recent AO Spine guidelines prioritize hemodynamic optimization as the only non-surgical intervention with potential benefit, while not recommending corticosteroids as part of standard management [25,66] Other contemporary guidelines also focus on early surgical decompression and intensive care management, without endorsing routine steroid therapy [67]. Taken together, these sources confirm that corticosteroid administration cannot be considered a general recommendation, as its efficacy remains uncertain and its use varies across clinical practice.

Surgical intervention was reserved for cases with clear cord compression by extraneural lesions (disc herniation or ligamentous injury seen on MRI), again reflecting the low intervention rates reported in the literature [54,62,63]. However, as highlighted in this review and notably in the case series of Liang et al. [63], surgical indications can be controversial: three cases described there involved sectioning the filum terminale after detecting a tight filum in children without pre-existing tethered cord syndrome manifestations, challenging the validity of filum surgery in such scenarios and reflecting a broader lack of consensus.

Essentially, even in observable extraneural MRI changes, the natural history can be variable; this review shows that many patients improve without surgical intervention. This highlights the call for more cautious, individualized surgical decision-making [25,37,53,63,65,67,68,69].

On the other hand, current clinical practice guidelines for acute SCI, including the most recent AO Spine recommendations, recommend early decompression and strict hemodynamic optimization as strategies to improve outcomes in adults [25,65,66]. Nevertheless, these guidelines are not specific to pediatric cases and are extrapolated from adult data, leaving uncertainty as to whether the timing of surgery and hemodynamic targets should be applied uniformly in children [66,67]. This lack of pediatric-focused recommendations underscores the need for further research and consensus to tailor management strategies for this distinct patient population.

### 4.4. Study Limitations and Future Directions

This systematic review offers a current overview of pediatric SCIWORA, compiling data from 848 patients across 60 studies. Major strengths include its rigorous PRISMA-based methodology, comprehensive aggregation of data, and detailed analysis of trauma mechanisms, MRI findings, and neurological outcomes. The review’s large sample size and stratified approach help identify both areas of consensus and ongoing clinical controversies, providing a practical reference for diagnosis and management.

However, several limitations should be acknowledged. There is significant heterogeneity among included studies regarding diagnostic criteria, MRI protocols, and follow-up durations. Most studies are retrospective, potentially introducing selection and reporting biases. Furthermore, the marked heterogeneity of the included studies, many of which were single case reports or small series, precluded the possibility of performing a formal meta-analysis. The wide variability in study design, sample size, and outcome reporting, and the absence of controlled or comparative studies on therapeutic strategies, therefore, limited quantitative synthesis, and our results are presented descriptively rather than statistically pooled. Additionally, evolving imaging technologies and a lack of standardized definitions contribute to inconsistencies across the published literature.

In summary, while this review offers robust and relevant insights, these inherent limitations and the need for further prospective, standardized research must be considered.

## 5. Conclusions

Pediatric SCIWORA remains a complex and diagnostically challenging entity characterized by diverse clinical presentations, varied trauma mechanisms, and heterogeneous MRI findings. MRI plays a crucial role in diagnosis and prognosis, distinguishing “real SCIWORA” (MRI-negative cases) from those with detectable abnormalities. While conservative management predominates and shows favorable outcomes in most patients, surgical interventions remain controversial and lack clear guidelines. This systematic review highlights the need for standardized diagnostic criteria, MRI classification schemes, and management protocols to optimize clinical outcomes in pediatric SCIWORA.

## Figures and Tables

**Table 1:** Patients’ demographics, clinical and radiological data. Trauma mechanism: sport/fall/RTA (road traffic accident); MRI types: I, no abnormalities; IIa, extraneural abnormalities; IIb, intraneural abnormalities; IIc, intraneural and extraneural abnormalities; Onset: negative sign (−): immediate onset; positive sign (+), delayed onset; Therapy: C, conservative; S, surgical; (S). NA, data not available.

Authors	Year	N.	Age	Sex		Trauma Mechanism				MRI Type				Onset	Therapy
				Male	Female	Sport	Fall	RTA	Other	I	IIa	IIb	IIc		
Pollack et al. [5]	1988	1	9	1	0	1	0	0	0	1	0	0	0	NA	NA
Matsumara et al. [2]	1990	1	3	0	1	0	0	1	0	0	0	1	0	+	C
Riviello et al. [6]	1990	2	2.5	0	2	0	1	0	0	0	1	1	0	+	C
Dickman et al. [15]	1991	26	10.5	19	7	7	7	12	0	5	0	2	1	NA	NA
Meuli et al. [16]	1991	1	5.3	0	1	0	0	0	1	1	0	0	0	NA	C
Ferguson et al. [26]	1993	1	2	1	0	0	0	1	0	0	0	1	0	−	C
Bondurant et al. [8]	1993	1	2.3	1	0	0	1	0	0	0	0	0	1	NA	C
Grabb et al. [9]	1994	7	7.4	5	2	0	0	1	2	1	2	3	1	NA	C
Felsberg et al. [27]	1995	12	9	8	4	1	0	9	0	7	0	3	2	NA	NA
Duprez et al. [10]	1998	1	2	1	0	0	1	0	0	0	0	0	1	+	NA
Pollina et al. [17]	1999	1	3	1	0	0	0	1	0	0	0	1	0	NA	C
Trumble et al. [7]	2000	1	3	0	1	0	0	1	0	0	0	1	0	+	C
Beck et al. [3]	2000	1	16	0	1	1	0	0	0	0	0	1	0	NA	C
Koestner et al. [28]	2001	1	1.4	0	1	0	0	0	1	0	0	0	1	+	C
Boockvar et al. [11]	2001	13	11.5	9	4	13	0	0	0	13	0	0	0	NA	C
Mortazavi et al. [29]	2001	1	1.8	1	0	0	1	1	0	0	0	0	1	NA	C
Yamaguchi et al. [12]	2002	1	14	0	1	0	1	0	0	0	0	0	1	+	C
Dare et al. [30]	2002	19	12.1	16	3	11	0	3	0	17	0	2	0	NA	C
Bosch et al. [4]	2002	9	5.7	6	3	0	2	5	2	0	1	8	0	+	C
Ergun et al. [31]	2003	1	12	0	1	1	1	1	0	0	0	1	0	NA	C
Liao et al. [32]	2005	9	4.1	6	3	0	4	5	0	3	0	6	0	NA	C
Lee et al. [33]	2006	1	1.2	1	0	0	1	0	0	1	0	0	0	+	C
Buldini et al. [18]	2006	2	5.7	1	1	0	1	1	0	0	0	1	1	+	C
Dickerman et al. [34]	2006	1	14	1	0	1	1	0	0	0	0	0	1	+	C
Kalra et al. [19]	2006	1	2.5	0	1	0	1	0	0	0	0	1	0	+	C
Rich et al. [35]	2006	1	5	\	0	\	0	0	0	0	1	0	0	−	S
Fregeville et al. [13]	2007	1	14	0	1	1	0	0	0	0	0	0	1	−	C
Shen et al. [36]	2007	1	6	0	1	1	1	0	0	0	0	1	0	NA	C
Kim et al. [23]	2008	1	1	0	1	0	1	0	0	0	0	1	0	+	C
Feldman et al. [37]	2008	2	0.3	0	2	0	1	0	0	0	0	1	1	+	C
Elmagal et al. [38]	2008	2	9	2	0	0	0	2	0	0	0	2	0	−	S
Grubenhoff et al. [20]	2008	1	7	1	0	1	0	0	0	0	0	1	0	NA	C
Silman et al. [39]	2008	1	0.9	1	0	0	0	1	0	0	0	0	1	−	C
Sullivan et al. [40]	2008	2	6	2	0	1	0	0	0	0	1	1	0	+	C
Triglylidas et al. [41]	2010	3	11.3	2	1	1	0	2	0	0	1	2	0	NA	C
Yalcin et al. [22]	2011	3	3.9	2	2	0	0	3	0	2	0	0	1	NA	2 C; 1 S
Snoek et al. [42]	2012	1	5	1	0	0	0	1	0	0	0	0	1	−	S
Abbo et al. [43]	2013	2	1.8	1	1	0	0	1	0	0	0	2	0	+	S
Phillips et al. [44]	2013	2	2	2	0	0	0	2	0	0	0	1	1	−	C
Mahajan et al. [45]	2013	69	11.1	52	17	27	18	8	16	54	0	15	0	NA	66 C, 3 S
Ayaz et al. [46]	2014	1	3.5	1	0	0	0	1	0	1	0	0	0	−	C
Fiaschi et al. [47]	2016	1	1.3	0	1	0	1	0	0	0	0	1	0	+	S
Knox et al. [48]	2016	297	10.66	191	106	122	41	76	58	Na	NA	NA	NA	NA	291 C, 6 S
Kim et al. [49]	2016	2	13.5	2	0	1	0	1	0	0	0	0	2	+	C
Bansal et al. [24]	2016	1	0.8	0	1	0	1	0	0	0	0	1	0	+	C
Nagasawa et al. [50]	2017	1	13	0	1	0	0	1	0	1	0	0	0	−	C
Jian et al. [51]	2017	12	6.6	0	12	12	0	0	0	0	10	0	2	+	C
Campbell et al. [52]	2018	1	2	0	1	0	0	1	0	0	0	1	0	−	C
Liang et al. [53]	2019	3	6.3	1	2	0	0	0	3	0	0	0	3	−	S
Bansal et al. [54]	2020	13	11	NA	NA	Na	NA	NA	NA	NA	NA	NA	NA	NA	NA
Brauge et al. [55]	2020	30	9.7	23	7	NA	NA	NA	NA	8	1	20	1	NA	29 C; 1 S
Kim et al. [56]	2021	1	7	1	0	0	0	1	0	0	0	0	1	−	C
Butts et al. [57]	2021	1	11	1	0	1	0	0	0	1	0	0	0	−	C
Liang et al. [53]	2022	16	6.3	1	15	12	4	0	0	1	0	15	0	−	C
Garcia-Cabra et al. [58]	2021	1	16	1	0	0	1	0	0	1	0	0	0	−	C
Freigang et al. [59]	2022	32	14.5	21	11	8	14	6	4	32	0	0	0	NA	C
Zou et al. [21]	2023	140	7.72	42	98	61	40	15	24	NA	NA	NA	NA	NA	C
Liu et al. [60]	2024	47	7.49	16	31	31	2	9	5	4	0	43	0	41−; 6+	1 S, 46 C
Hu et al. [61]	2024	39	7.2	3	36	25	10	4	0	1	6	34	6	NA	NA
Romero-Munoz et al. [62]	2024	13	4	10	3	1	1	10	1	2	0	7	4	12−/1+	12 C; 1 S
Total		848	9.33 ± 2.52	458	388	342	159	187	117	157	24	182	36		801 C, 39 S, 8 NA

**Table 2:** ASIA grading at admission and discharge.

Authors	Year	N.	Location	Worst ASIA	ASIA Final FUP	% of Improvement	% of Stability
Pollack et al. [5]	1988	1	1 CT	C	E	100%	0%
Matsumara et al. [2]	1990	1	1 CT	A	A	0%	100%
Riviello et al. [6]	1990	2	1 C 1 CT	2 B	1 C 1 D	100%	0%
Dickman et al. [15]	1991	26	18 C 8 T	7 A 2 B 5 C 12 D 0 E	2 A 0 B 2 C 3 D 17 E 2 Died	80%	20%
Meuli et al. [16]	1991	1	1 T	C	E	100%	0%
Ferguson et al. [26]	1993	1	1 T	A	A	0%	100%
Bondurant et al. [8]	1993	1	1 C	B	C	100%	0%
Duprez et al. [10]	1998	1	1 CT	A	A	100%	0%
Pollina et al. [17]	1999	1	1 T	A	A	0%	100%
Trumble et al. [7]	2000	1	1 T	A	A	0%	100%
Beck et al. [3]	2000	1	1 C	A	E	100%	0%
Koestner et al. [28]	2001	1	1 High C	B	B	0%	100%
Boockvar et al. [11]	2001	13	1 C	7 C 5 D	13 E	100%	0%
Mortazavi et al. [29]	2001	1	1 T	B	B	0%	100%
Yamaguchi et al. [12]	2002	1	1 T	B	D	100%	0%
Dare et al. [30]	2002	19	NA	2 A 0 B 1 C 16 D 0 E	1 A 1 B 0 C 2 D 15 E	95%	5%
Bosch et al. [4]	2002	9	NA	5 A 1 B 1 C 1 D 1 E	5 A 1 B 1 C 2 E	11%	88%
Ergun et al. [31]	2003	1	1 CT	A	C	100%	0%
Liao et al. [32]	2005	9	2 C 2 CT 5 T	3 A 1 B 2 C 3 D	2 A 3 C 1 D 3 E	66%	33%
Lee et al. [33]	2006	1	None	C	E	100%	0%
Buldini et al. [18]	2006	2	1 CT 1 T	2 A	A, C	50%	50%
Dickerman et al. [34]	2006	1	1 C	C	E	100%	0%
Kalra et al. [19]	2006	1	1 C	A	A	0%	100%
Rich et al. [35]	2006	\	1 C	D	E	100%	0%
Fregeville et al. [13]	2007	1	1 High C	A	C	100%	0%
Shen et al. [36]	2007	1	T	C	D	100%	0%
Kim et al. [23]	2008	1	1 Low C	C	D	100%	0%
Feldman et al. [37]	2008	2	2 C	1 A 1 C	1 A 1 E	50%	50%
Elmagal et al. [38]	2008	1	1 C	A	A	50%	50%
Grubenhoff et al. [20]	2008	1	1 C	B	C	100%	0%
Silman et al. [39]	2008	1	1 CT	A	A	0%	100%
Sullivan et al. [40]	2008	2	1 C 1 T	A D	A E	50%	50%
Triglylidas et al. [41]	2010	3	1 C 2 T	2 A 1 D	2 A 1 E	33%	66%
Yalcin et al. [22]	2011	3	3 T 1 L	2 A 1 B	1 A 2 C	66%	33%
Snoek et al. [42]	2012	1	1 C	B	E	100%	0%
Abbo et al. [43]	2013	2	1 CT, 1 TL	2 A	1 C, 1 E	100%	0%
Phillips et al. [44]	2013	2	1 T, 1 CT	2 A	1 A, 1 B	50%	50%
Mahajan et al. [45]	2013	69	69 C	NA	NA	NA	NA
Ayaz et al. [46]	2014	1	1 T	A	C	100%	0%
Fiaschi et al. [47]	2016	1	1 High C	E	E	0%	100%
Knox et al. [48]	2016	297	137 C 41 T 21 L 1 S 150 NA	NA	NA	NA	NA
Kim et al. [49]	2016	2	1 Low CA 1 T	1 A 1 D	1 A 1 E	50%	50%
Bansal et al. [24]	2016	1	1 C	A	C	100%	0%
Nagasawa et al. [50]	2017	1	1 C	D	E	100%	0%
Jian et al. [51]	2017	12	5 T 6 TL 1 CT	10 A 2 B	7 A 2 B 2 C 0 D 1 E	41.60%	58.40%
Campbell et al. [52]	2018	1	1 T	A	A	0%	100%
Liang et al. [53]	2019	3	2 TL, 1 T	3 A	3 A	0%	100%
Bansal et al. [54]	2020	13	8 T 4 C 1 L	6 A 1 B 1 D 5 E	NA	NA	NA
Brauge et al. [55]	2020	30	19 C 10 T 1 L	14 A 3 B 9 C 3 D 1 E	2 died 5 A 3 B 1 C 19 E	66.70%	33.30%
Kim et al. [56]	2021	1	1 High C	C	D	100%	0%
Butts et al. [57]	2021	1	None	E	E	0%	100%
Liang et al. [53]	2021	16	2 CT 14 C	7 A 3 B 3 C 3 D 0 E	6 A 0 B 3 C 3 D 4 E	62.50%	37.50%
Garcia-Cabra et al. [58]	2021	1	1 C	A	C	100%	0%
Freigang et al. [59]	2022	32	NA	6 A 3 B 12 C 11 D 0 E	32 E	100%	0%
Zou et al. [21]	2023	140	14 C	70 A 24 B 20 C 20 D 1 E 5 NA	NA	NA	NA
Liu et al. [60]	2024	47	9 C 6 CT 22 T 8 TL 2 L	28 A 13 B 5 C 1 D 0 E	24 A 12 B 8 C 2 D 1 E	21.20%	79.80%
Hu et al. [61]	2024	39	4 C 26 T 9 L	20 A 5 B 4 C 10 D 0 E	NA	43.60%	56%
Romero-Munoz et al. [62]	2024	13	7 C 6 T	4 A 4 B 4 C 1 D 0 E	4 A 3 B 2 C 4 D 0 E	23%	77%
Total		848	331 C (58.79%) 155 T (27.53%) 36 L (6.39%) 23 CT (4.08%) 17 TL (3.01%) 1 S (0.17%)	A 210 (46.25%) B 70 (15.41%) C 77 (16.96%) D 87 (19.16%) E 10 (4.54%)	A 56 (24.45%) B 14 (6.11%) C 26 (11.35%) D 19 (8.29%) E 114 (49.78%)	66.2%	33.8%

**Table 3:** Operative cases and surgery type.

Author	Year	N.	Type of Lesion	Type of Surgery
Rich et al. [35]	2006	1	cervical	anterior cervical decompression and fusion using iliac crest bone graft and plate
Yalcin et al. [22]	2011	1	Lumbar	Laminectomy
Snoek et al. [42]	2012	1	Multiple	Decompression of the spinal cord and fusion of the second and third vertebral bodies (L2–L3)
Mahajan et al. [45]	2013	3	Cervical	Internal fixation/halo
Fiaschi et al. [47]	2016	1	C1C3 hematomyelia	Occipito-cervical (C1) decompression
Knox et al. [48]	2016	6	NA	NA
Liang et al. [63]	2019	3	T9L1 with tight filum terminale T12L1 with tight filum terminale T6T10 with tight filum terminale	lysis of the filum terminale lysis of the filum terminale lysis of the filum terminale
Brauge et al. [55]	2020	1	Cervical	Surgery for cervical diastemtomyelia
Liu et al. [60]	2024	1	NA	NA
Romero Munoz et al. [62]	2024	1	Cervical	Laminectomy for posterior decompression

## Data Availability

All data supporting the findings of this study are available within the paper.

## Supplementary Materials

The following supporting information can be downloaded at https://www.mdpi.com/article/10.3390/jcm14176338/s1, Table S1: JBI critical appraisal tool for case series’ risk of bias assessment; Table S2: JBI critical appraisal tool for case reports’ risk of bias assessment.

## Abbreviations

The following abbreviations are used in this manuscript:

SCIWORA	Spinal Cord Injury Without Radiographic Abnormality
MRI	Magnetic Resonance Imaging
CT	Computed Tomography
ASIA	American Spinal Injury association Impairment Scale
RTA	Road Traffic Accident
CNS	Central Nervous System
