# Supplementary material for: Redefining Pediatric SCIWORA: A Systematic Review of the Literature on Clinical Patterns, Imaging Profiles, and Management Insights
Source: J Clin Med. 2025 Sep 8;14(17):6338. doi: 10.3390/jcm14176338 (PMC12429215; doi:10.3390/jcm14176338)
Supplement: Supplementary file 1 [file jcm-14-06338-s001.zip › jcm-3826804-supplementary.pdf]

Journal of Clinical Medicine

| | Riviello et al. [6] | Dickman et al. [15] | Grabb et al. [9] | Felsberg et al. [27] | Boockvar et al. [11] | Dare et al. [30] | Bosch et al. [4] | Liao et al. [21] | Buldini et al. [18] | Feldman et al. [37] | Elmagal et al. [38] | Sullivan et al. [40] | Triglylidas et al. [41] | Yalcin et al. [22] |
|---|---|---|---|---|---|---|---|---|---|---|---|---|---|---|
| Q1 | Y | Y | Y | Y | Y | Y | Y | Y | Y | Y | Y | Y | Y | Y |
| Q2 | Y | Y | Y | Y | Y | Y | Y | Y | Y | Y | Y | Y | Y | Y |
| Q3 | Y | Y | Y | Y | Y | Y | Y | Y | Y | Y | Y | Y | Y | Y |
| Q4 | Y | Y | Y | Y | Y | Y | Y | Y | Y | Y | Y | Y | Y | Y |
| Q5 | Y | Y | Y | Y | Y | Y | Y | Y | Y | Y | Y | Y | Y | Y |
| Q6 | Y | Y | Y | Y | Y | Y | Y | Y | Y | Y | Y | Y | Y | Y |
| Q7 | Y | Y | U | U | Y | Y | Y | Y | Y | Y | Y | Y | Y | Y |
| Q8 | Y | Y | U | U | Y | Y | Y | Y | Y | Y | Y | Y | Y | Y |
| Q9 | Y | N | N | N | U | U | Y | Y | Y | Y | U | Y | U | U |
| Q10 | NA | NA | NA | NA | NA | NA | NA | NA | NA | NA | NA | NA | NA | NA |

Supplementary Table S1: JBI critical appraisal tool for case series' risk of bias assessment (continues on the next page).

 https://doi.org/10.3390/10.3390/jcm14176338

| | Abbo et al. [43] | Phillips et al. [44] | Mahajan et al. [45] | Knox et al. [48] | Kim et al. [49] | Jian et al. [51] | Liang et al. [53] | Bansal et al. [54] | Brauge et al. [55] | Liang et al. [53] | Freigang et al. [59] | Zou et al. [21] | Liu et al. [60] | Hu et al. [61] | Romero-Munoz et al. [62] |
|---|---|---|---|---|---|---|---|---|---|---|---|---|---|---|---|
| Q1 | Y | Y | Y | Y | Y | Y | Y | Y | Y | Y | Y | Y | Y | Y | Y |
| Q2 | Y | Y | Y | Y | Y | Y | Y | Y | Y | Y | Y | Y | Y | Y | Y |
| Q3 | Y | Y | Y | Y | Y | Y | Y | Y | Y | Y | Y | Y | Y | Y | Y |
| Q4 | Y | Y | Y | Y | Y | Y | Y | Y | Y | Y | Y | Y | Y | Y | Y |
| Q5 | Y | Y | Y | Y | Y | Y | Y | Y | Y | Y | Y | Y | Y | Y | Y |
| Q6 | Y | Y | Y | Y | Y | Y | Y | Y | Y | Y | Y | Y | Y | Y | Y |
| Q7 | Y | Y | N | U | Y | Y | Y | U | Y | Y | Y | Y | Y | U | Y |
| Q8 | Y | Y | N | U | Y | Y | Y | U | Y | Y | Y | Y | Y | U | Y |
| Q9 | Y | U | N | U | Y | Y | Y | Y | U | Y | Y | Y | Y | U | Y |
| Q10 | NA | NA | NA | NA | NA | NA | NA | NA | NA | NA | Y | NA | NA | Y | NA |

| | Pollack et al. [5] | Matsumara et al. [2] | Meuli et al. [16] | Ferguson et al. [26] | Bondurant et al. [8] | Duprez et al. [10] | Pollina et al. [17] | Trumble et al. [7] | Beck et al. [3] | Koestner et al. [28] | Mortazavi et al. [29] | Yamaguchi et al. [12] | Ergun et al. [31] | Lee et al. [33] | Dickerman et al. [34] |
|---|---|---|---|---|---|---|---|---|---|---|---|---|---|---|---|
| Q1 | Y | Y | Y | Y | Y | Y | Y | Y | Y | Y | Y | Y | Y | Y | Y |
| Q2 | Y | Y | Y | Y | Y | Y | Y | Y | Y | Y | Y | Y | Y | Y | Y |
| Q3 | Y | Y | Y | Y | Y | Y | Y | Y | Y | Y | Y | Y | Y | Y | Y |
| Q4 | Y | Y | Y | Y | Y | Y | Y | Y | Y | Y | Y | Y | Y | Y | Y |
| Q5 | U | N | N | N | N | N | Y | Y | Y | Y | Y | Y | Y | Y | Y |
| Q6 | Y | Y | Y | Y | Y | Y | Y | Y | Y | Y | Y | Y | Y | Y | Y |
| Q7 | NA | NA | NA | NA | NA | NA | NA | NA | NA | NA | NA | NA | NA | NA | NA |
| Q8 | Y | Y | Y | Y | Y | Y | Y | Y | Y | Y | Y | Y | Y | Y | Y |

Supplementary Table S2: JBI critical appraisal tool for case reports’ risk of bias assessment (continues on the next page).

| | Kalra et al. [19] | Rich et al. [35] | Fregeville et al. [13] | Silman et al. [39] | Grubenhoff et al. [20] | Kim et al. [23] | Shen et al. [36] | Snoek et al. [42] | Ayaz et al. [46] | Fiaschi et al. [47] | Bansal et al [24] | Nagasawa et al [50] | Campbell et al. [52] | Kim et al. [56] | Butts et al. [57] | Garcia-Cabra et al. [58] |
|---|---|---|---|---|---|---|---|---|---|---|---|---|---|---|---|---|
| Q1 | Y | Y | Y | Y | Y | Y | Y | Y | Y | Y | Y | Y | Y | Y | Y | Y |
| Q2 | Y | Y | Y | Y | Y | Y | Y | Y | Y | Y | Y | Y | Y | Y | Y | Y |
| Q3 | Y | Y | Y | Y | Y | Y | Y | Y | Y | Y | Y | Y | Y | Y | Y | Y |
| Q4 | Y | Y | Y | Y | Y | Y | Y | Y | Y | Y | Y | Y | Y | Y | Y | Y |
| Q5 | Y | Y | Y | Y | Y | Y | Y | N | N | N | Y | Y | Y | Y | U | U |
| Q6 | Y | Y | Y | Y | Y | Y | Y | Y | Y | Y | Y | Y | Y | Y | Y | Y |
| Q7 | NA | NA | NA | NA | NA | NA | NA | NA | NA | NA | NA | NA | NA | NA | NA | NA |
| Q8 | Y | Y | Y | Y | Y | Y | Y | Y | Y | Y | Y | Y | Y | Y | Y | Y |
